# Health care use and spending for Medicaid patients diagnosed with opioid use disorder receiving primary care in Federally Qualified Health Centers and other primary care settings

**DOI:** 10.1371/journal.pone.0276066

**Published:** 2022-10-18

**Authors:** Lauren Peterson, Manoradhan Murugesan, Robert Nocon, Hank Hoang, Joshua Bolton, Neda Laiteerapong, Harold Pollack, Jeanne Marsh

**Affiliations:** 1 Crown Family School of Social Work, Policy, and Practice, University of Chicago, Chicago, Illinois, United States of America; 2 Department of Public Health Sciences, University of Chicago, Chicago, IL, United States of America; 3 Department of Health Systems Science, Kaiser Permanente Bernard J. Tyson School of Medicine, Pasadena, California, United States of America; 4 Health Resources and Services Administration, U.S. Department of Health and Human Services, Rockville, Maryland, United States of America; 5 Department of Medicine, University of Chicago, Chicago, Illinois, United States of America; Sorbonne University Pierre and Marie Curie Campus: Sorbonne Universite Campus Pierre et Marie Curie, FRANCE

## Abstract

**Introduction:**

This nationwide study builds on prior research, which suggests that Federally Qualified Health Centers (FQHCs) and other primary care providers are associated with increased access to opioid use disorder (OUD) treatment. We compare health care utilization, spending, and quality for Medicaid patients diagnosed with OUD who receive primary care at FQHCs and Medicaid patients who receive most primary care in other settings, such as physician offices (non-FQHCs). We hypothesized that the integrated care model of FQHCs would be associated with greater access to medication for opioid use disorder (MOUD) and/or behavioral health therapy and lower rates of potentially inappropriate co-prescribing.

**Methods:**

This cross-sectional study examined 2012 Medicaid Analytic eXtract files for patients diagnosed with OUD receiving most (>50%) primary care at FQHCs (N = 37,142) versus non-FQHCs (N = 196,712) in all 50 states and Washington DC. We used propensity score overlap weighting to adjust for measurable confounding between patients who received care at FQHCs versus non-FQHCs and increase generalizability of findings given variation in Medicaid programs and substance use policies across states.

**Results:**

FQHC patients displayed higher primary care utilization and fee-for-service spending, and similar or lower utilization and fee-for-service spending for other health service categories. Contrary to our hypotheses, non-FQHC patients were more likely to receive timely (≤90 days) MOUD (buprenorphine, methadone, naltrexone, or suboxone) (Relative Risk [RR] = 1.10, 95% CI: 1.07, 1.12) and more likely be retained in medication treatment (>180 days) (RR = 1.12, 95% CI: 1.09, 1.14). However, non-FQHC patients were less likely to receive behavioral health therapy (mental health or substance use therapy) (RR = 0.90, 95% CI: 0.88, 0.92) and less likely to remain in behavioral health treatment (RR = 0.92, 95% CI: 0.89, 0.94). Non-FQHC patients were more likely to fill potentially inappropriate prescriptions of benzodiazepines and opioids after OUD diagnosis (RR = 1.35, 95% CI: 1.30, 1.40).

**Conclusions:**

Observed patterns suggest that Medicaid patients diagnosed with OUD who obtained primary care at FQHCs received more integrated care compared to non-FQHC patients. Greater care integration may be associated with increased access to behavioral health therapy and quality of care (lower potentially inappropriate co-prescribing) but not necessarily greater access to MOUD.

## Introduction

Drug overdose mortality quadrupled in the United States between 1999 and 2019, largely due to the opioid epidemic [1]. By 2017, 38% of non-elderly adults diagnosed with an opioid use disorder (OUD) were covered by Medicaid, reflecting the central role of this public insurance program in addressing the crisis [2]. Medicaid has become the largest source of financing for OUD treatment and provides coverage for medication for opioid use disorder (MOUD), the first-line standard of care, in addition to other pertinent behavioral health therapies [2–5].

Despite the expansion in Medicaid coverage and funding, less than half of non-elderly adult Medicaid patients diagnosed with OUD received treatment in 2017 [2]. Improving access to primary care has become a key policy strategy to increase OUD treatment utilization [6]. Primary care has been associated with greater MOUD utilization among Medicaid patients [7]. Analyses of Pennsylvania Medicaid fee-for-service claims data indicate that patients diagnosed with OUD who had at least one primary care visit were more likely to receive MOUD and more likely to fill medication treatment prescriptions as compared to patients diagnosed with OUD and no primary care visits [6].

Research further suggests that a multidisciplinary, coordinated care model can improve OUD treatment [8]. Many Federally Qualified Health Centers (FQHCs) follow an integrated approach to comprehensive primary care. Section 330 of the Public Health Service Act requires Health Resources and Services Administration-funded FQHCs to provide an array of required primary health services to enable holistic and patient-centered care to some of the most underserved populations. These services include, but are not limited to, preventive health services, referral to substance use disorder and mental health services, patient case management services, and enabling services. Prior research demonstrates a positive association between integrated buprenorphine maintenance treatment provided by FQHCs and treatment retention in Connecticut [9]. Integration of primary and behavioral health services within FQHCs has also been associated with improved OUD treatment access and retention in care for Medicaid patients in Pennsylvania [10].

While these studies provide preliminary evidence of the effectiveness of Medicaid and primary care in improving OUD treatment access and retention in care, the current literature largely focuses on a few states and narrow explorations of specific MOUD treatments, with limited consideration of different types of primary care settings. Research is also needed to characterize the population of Medicaid patients diagnosed with OUD who receive primary care, MOUD, and behavioral health therapies at FQHCs and other outpatient care settings, and to explore utilization and quality of such services across the United States [4, 5, 10–12].

In this study, we used calendar-year 2012 Medicaid claims data from all fifty states and Washington DC to compare demographic characteristics, health care utilization, and health care spending for Medicaid patients diagnosed with OUD who received the majority of their primary care services in FQHCs versus non-FQHCs, such as hospital outpatient clinics and physician offices. While the opioid epidemic has intensified and evolved since 2012, our data enabled us to examine how primary care setting, particularly the integrated care model used by many FQHCs, may affect OUD treatment access, retention, and quality among a nationally representative sample of Medicaid patients diagnosed with OUD. The 2012 Medicaid claims data also provide greater consistency and uniformity for national-level analyses compared to more recent Medicaid claims data, which have greater variation across and within states, especially across managed care plans.

Considering the integrated care model adopted by many FQHCs, we hypothesized that Medicaid patients diagnosed with OUD who received the majority of their primary care in FQHCs (FQHC patients) would be more likely to receive MOUD or behavioral health therapy, and to receive more timely access and greater retention in treatment, as compared to Medicaid patients who received most of their primary care in other settings (non-FQHC patients). We also hypothesized that FQHC patients would be less likely to exhibit potentially inappropriate concurrent prescribing, such as opioid analgesics with benzodiazepines, following OUD diagnosis. Finally, drawing from the findings of Nocon et al. (2016), we anticipated that FQHC patients would have lower health care utilization and health care spending, as compared to non-FQHC patients.

## Methods

### Study design

We examined the cross-sectional association between primary care setting (FQHC versus non-FQHC) and utilization, spending, and quality outcomes for Medicaid patients diagnosed with OUD in all 50 states and the District of Columbia. We used the propensity score-based overlap weighting method to adjust for measurable confounding between patients who received primary care at FQHCs versus non-FQHCs. The study was approved by the University of Chicago Biological Sciences Division Institutional Review Board (IRB). The IRB determined the study met the standard for waiver of consent.

### Data sources and study population

We used 2012 Medicaid Analytic eXtract (MAX) files and restricted the study population to non-elderly adults (aged 18–64 years) with an OUD diagnosis, at least one primary care visit, and Medicaid enrollment for at least six continuous months following OUD diagnosis in calendar year 2012. We included individuals enrolled in fee-for-service Medicaid or Medicaid managed care programs. We excluded patients with restricted benefits and Medicaid-Medicare dual eligible patients.

Building on prior research by Young and Zur (2017), we identified individuals diagnosed with OUD using ICD-9 codes for opioid dependence (304.x), opioid abuse (305.x), and opioid poisoning (965.x) [13]. (See S1 File, Data Availability Statement and Construction, for further description.) Most patients in our sample were diagnosed with opioid dependence (304.x). We found some claims with invalid ICD-9 codes that appeared to be ICD-10 codes. We treated this as a data quality issue and dropped all Medicaid patients with such claims from the sample.

We studied both general health care and OUD treatment utilization outcomes. General health care expenditure and utilization outcomes included spending (cost) and counts for primary care, other outpatient, inpatient, addiction treatment, and emergency department visits, and prescriptions filled by the patient. Spending represented the sum of total payments from Medicaid and third-party payers. Timely receipt of OUD treatment, retention in care, and concurrent opioid analgesic and benzodiazepine prescriptions filled comprised our OUD treatment outcomes.

Based on prior research, we used a time-window of ninety days from OUD diagnosis to construct timely receipt of MOUD and/or behavioral health therapy [14]. Building on research by Cole et al. (2019) and the Medicaid Outcomes Distributed Research Network (2021), we used a time-window of greater than 180 days for retention in care. We further validated these time-window selections with sensitivity analyses with 1, 2, 3, and 6-month time-windows. (See S4 File, Sensitivity Analysis, for results.)

We defined MOUD as one or more filled prescriptions or claims for the following OUD medications approved by the Food and Drug Administration (FDA): buprenorphine, suboxone, methadone, and naltrexone. We used pharmacy claims to identify buprenorphine (without naloxone), suboxone, and naltrexone treatments with the aid of an NDC list compiled using National Library of Medicine’s RxNorm API. We used Healthcare Common Procedure Coding System (HCPCS) codes to identify methadone and oral or long-acting injectable naltrexone.

Building on prior literature, we also used NDC codes to identify benzodiazepines and opioid analgesics to examine co-prescribing [4, 15]. We defined behavioral health therapy as one or more claims for substance use intervention therapy and mental health support. Our definition of behavioral health therapy was based on services that fall under outpatient OUD treatment in the existing literature [5, 6, 14]. (See S1 File, Data Availability Statement and Construction, for listings of these codes and variable construction details.)

Primary care setting was our main independent variable of interest. Primary care visits were identified using a combination of evaluation and management codes, provider taxonomies, and claim setting or type of service [16]. We identified primary care setting by creating a list of FQHC identifiers from the Health Resources and Services Administration (HRSA) databases as well as Medicare and Medicaid cost reports. We linked these data to the National Plan and Provider Enumeration System to obtain provider identifications present in claims. We categorized FQHC patients as those with more than half of primary care visits occurring at a FQHC. Individuals with no more than half of all primary care visits occurring at a FQHC, or with no FQHC primary care visits, were categorized as non-FQHC patients (hospital outpatient, physician office, or a mix).

Our adjusted analysis included patient demographics, insurance characteristics, disease burden, and state of residence as covariates to account for factors that may increase health care access, utilization, and spending.

### Statistical analysis

We reported all descriptive statistics in counts (percentages) for binary variables and means (standard deviations) for continuous variables. Outcomes comparisons for statistically significant differences between primary care settings were developed using generalized estimated equation (GEE) model estimates. We reported the differences in terms of relative risks (RR) and incident rate ratio (IRR). RR and IRR ratios compare the adjusted non-FQHC estimate to the adjusted FQHC estimate. To transparently compare across non-FQHC care settings, a value of less than one reflects lower non-FQHC utilization or spending and a value of greater than one reflects higher non-FQHC utilization or spending.

We used the propensity score-based overlap weighting method to balance covariates in each of the primary care groups. Multivariate logistic regression modeling with these covariates was used to estimate propensity scores for primary care setting assignment. Balanced primary care setting samples were then created by assigning the probability of assignment in a primary care setting that is different from their actual assignment as weights to each patient. Comparison of covariate values between settings with these weights showed that all samples were balanced with respect to the selected confounders (See S2 File, Propensity Overlap Weighting Balancing Table).

We then modeled our outcomes by using GEE method for patients in the balanced samples with weights. We used the log link function with negative binomial distribution to model counts outcomes, such as number of primary care visits, and the logit link function to model probability of each category for binary outcomes. Count outcomes with an excess of zero counts, such as number of inpatient days and visits were dichotomized into binary data (yes or no service use) and then modeled by GEE for binary outcome analysis. We conducted sensitivity/sub-group analyses using the same analysis methods to check the consistency of our main findings and identify any heterogeneity in our data.

Covariates and outcomes were constructed from raw claims using Python (3.8) and all statistical analysis was performed using SAS 9.4 (SAS Institute, Cary, NC). All of our p-values are two-tailed with a significance threshold of 0.05.

## Results

### Demographics

The analytic sample included 37,142 FQHC and 196,712 non-FQHC patients with at least six months of Medicaid eligibility after an OUD diagnosis. As shown in Table 1, on average, FQHC patients were 42 years of age, and non-FQHC patients were 40 years of age. FQHC patients were less-often female as compared to non-FQHC patients (FQHC = 50.4% versus non-FQHC = 58.0%). Using the race and ethnicity categories specified in the Medicaid MAX files, a higher proportion of FQHC patients were identified as non-Hispanic Black (FQHC = 19.8% versus non-FQHC = 14.3%) or Hispanic (FQHC = 18.5% versus non-FQHC = 11.6%) and a lower proportion of FQHC patients were identified as non-Hispanic white (FQHC = 52.9% versus non-FQHC = 65.6%) as compared to non-FQHC patients. The identified proportions of Asian (FQHC = 0.5% versus non-FQHC = 0.5%), American Indian/Alaska Native (FQHC = 1.3% versus non-FQHC = 1.3%), Native Hawaiian/Other Pacific Islander (FQHC = 0.3% versus non-FQHC = 0.2%), and multi-racial (FQHC = 0.4% versus non-FQHC = 0.5%) patients were similar across both settings. Race or ethnicity data was missing for 6.3% of FQHC patients and 6.0% of non-FQHC patients.

**Table 1:** Characteristics of patients with at least six months of continuous Medicaid enrollment after OUD diagnosis by primary care setting: United States, 2012.

Characteristic	FQHC, N (%) or Mean ± SD	Non-FQHC, N (%) or Mean ± SD			
		Combined Non-FQHC	Hospital Outpatient	Physician Office	Mixed Use
Patients (N = 233854)	37142	196712	51053	114940	30719
Age (years)	42.4 ± 11.5	39.8 ± 11.5	41.3 ± 11.9	38.7 ± 11.3	38.1 ± 11.4
Female	(50.4)	(58.0)	(48.8)	(61.1)	(61.2)
Race/ethnicity					
*White*	(52.9)	(65.6)	(52.8)	(70.0)	(70.1)
*Black*	(19.8)	(14.3)	(18.4)	(13.1)	(11.9)
*Hispanic/Latino*	(18.5)	(11.6)	(21.0)	(8.1)	(9.3)
*Asian or Pacific Islander*	(0.5)	(0.5)	(0.7)	(0.4)	(0.3)
*American Indian or Alaska Native*	(1.3)	(1.3)	(1.4)	(1.2)	(1.7)
*Native Hawaiian or Other Pacific Islander*	(0.3)	(0.2)	(0.1)	(0.2)	(0.1)
*More than One Race*	(0.4)	(0.5)	(0.8)	(0.4)	(0.5)
*Missing*	(6.3)	(6.0)	(4.7)	(6.5)	(6.1)
Dominant Medicaid eligibility group					
*Blind/disabled*	(40.9)	(34.8)	(36.6)	(34.4)	(33.1)
*Adult*	(36.4)	(40.8)	(26.2)	(45.8)	(46.7)
*State demonstration*	(22.6)	(24.4)	(37.2)	(19.8)	(20.2)
Medicaid eligible months	11.6 ± 1.1	11.6 ± 1.2	11.5 ± 1.2	11.6 ± 1.2	11.5 ± 1.2
Maximum consecutive months of Medicaid enrollment	11.6 ± 1.2	11.5 ± 1.3	11.5 ± 1.3	11.6 ± 1.2	11.5 ± 1.3
Medicaid managed care months	6.2 ± 5.5	8.1 ± 5.0	7.4 ± 5.1	8.5 ± 4.9	7.8 ± 5.1
Medicaid fee-for-service months	5.4 ± 5.5	3.4 ± 4.9	4.0 ± 4.9	3.0 ± 4.7	3.7 ± 5.0
Months after OUD diagnosis	10.2 ± 2.1	10.1 ± 2.1	10.3 ± 2.1	10.2 ± 2.1	10.2 ± 2.1
Census Region					
*Midwest*	(10.1)	(20.4)	(13.6)	(22.9)	(22.3)
*Northeast*	(53.2)	(45.9)	(73.1)	(35.4)	(39.7)
*South*	(13.3)	(20.0)	(7.8)	(25.3)	(20.4)
*West*	(23.4)	(13.8)	(5.6)	(16.4)	(17.7)
Urban	(89.5)	(82.6)	(86.8)	(81.6)	(79.1)
Minimum distance from FQHC (km)	10.4 ± 15.4	18.4 ±23.6	13.2 ± 20.7	20.5 ± 24.9	18.8 ± 21.7
TANF eligible	(6.2)	(6.4)	(5.3)	(6.6)	(7.3)
CDPS risk score	2.5 ± 1.9	2.7 ± 2.0	2.9 ± 2.3	2.6 ± 2.0	2.5 ± 1.9
Elixhauser risk score	3.6 ± 2.3	3.5 ± 2.5	3.7 ± 2.7	3.4 ± 2.4	3.6 ± 2.2

FQHC patients further differed from non-FQHC patients on several covariates, including dominant Medicaid eligibility on the basis of a disability (FQHC = 40.9% versus non-FQHC = 34.8%), urbanicity (FQHC = 89.5% versus non-FQHC = 82.6%), and average distance from the nearest FQHC (FQHC = 10.4 km versus non-FQHC = 18.4 km). A majority of FQHC patients resided in the Northeast (53.2%) with the remainder of FQHC patients living in the West (23.4%), the South (13.3%), and the Midwest (10.1%). A smaller percentage of non-FQHC patients lived in the Northeast (45.9%) with the remainder of non-FQHC patients residing in the Midwest (20.4%), the South (20.0%), and the West (13.8%). All described differences between groups were statistically significant (p<0.05) due to large population size. After matching using the propensity score-based overlap weighing method, the FQHC and non-FQHC groups were equivalent on all demographic characteristics (see S2 File, Propensity Overlap Weighting Balancing Table).

### Health care utilization and spending

Before introducing propensity score-based overlap weighting (as shown in S3.1 Table in S3 File, Unadjusted Analyses), on average, patients who received most of their primary care from FQHCs had more primary care visit claims (FQHC = 17.8 versus non-FQHC = 16.1) and addiction treatment visit claims (FQHC = 24.1 versus non-FQHC = 21.6) and the same or fewer visits for emergency department care (FQHC = 3.2 versus non-FQHC = 3.2), inpatient care (FQHC = 1.0 versus non-FQHC = 1.0), other outpatient care (non-primary care, non-transportation services, and non-dental outpatient claims) (FQHC = 47.9 versus non-FQHC = 46.5), and filled prescriptions (FQHC = 45.8 versus non-FQHC = 47.1). FQHC patients who were enrolled in a fee-for-service plan for all Medicaid eligible months also had higher mean primary care spending (FQHC = $2,792 versus non-FQHC = $1,979) and mean addiction treatment spending (FQHC = $1,617 versus non-FQHC = $1,143) and similar or lower mean spending for emergency department care (FQHC = $1,241 versus non-FQHC = $1,344), inpatient care (FQHC = $5,099 versus non-FQHC = $6,423), other outpatient care (FQHC = $3,712 versus non-FQHC = $4,064), prescription drugs (FQHC = $4,279 versus non-FQHC = $4,543), and total health care (FQHC = $17,124 versus non-FQHC = $18,353). Fee-for-service (FFS) spending estimates only include patients with fee-for-service coverage for all eligible months due to concerns with quality of managed care expenditures data in some states. The fee-for-service population includes 14,708 FQHC patients and 44,820 non-FQHC patients.

As shown in Table 2, these comparisons were adjusted using the propensity score-based overlap weighting method to balance covariates. RR and IRR ratios compare the adjusted non-FQHC estimate to the adjusted FQHC estimate, with a value of less than one reflecting lower non-FQHC utilization or spending. FQHC patients continued to show higher primary care utilization (RR = 0.81, 95% CI: 0.81, 0.82) and fee-for-service spending (IRR = 0.70, 95% CI: 0.69, 0.71) and similar or lower mean utilization for emergency department care (RR = 0.98, 95% CI: 0.97, 0.99), inpatient care (RR = 1.00, 95% CI: 0.99, 1.02), other outpatient care (RR = 1.05, 95% CI: 1.04, 1.06), and prescription drugs (RR = 1.04, 95% CI: 1.03, 1.05). After adjusting using the propensity score-based overlap weighting method, FQHC patients also had lower addiction treatment use (RR = 1.08, 95% CI: 1.06, 1.10).

**Table 2:** Annual use and spending for FQHC OUD patients compared with non-FQHC OUD patients with at least six methods of continuous Medicaid enrollment after OUD diagnosis: United States, 2012.

Annual Utilization or Spending	Adjusted FQHC Estimate (95% CI)	Adjusted Non-FQHC Estimate (95% CI)	Adjusted RR / IRR, (95% CI)
Emergency department	(65.7)	(65.0)	0.99 (0.98, 1.00)
*Visits (N)*	3.0 (3.0, 3.0)	3.0 (2.9. 3.0)	0.98 (0.97, 0.99)
*FFS Sample Spending ($)*	1252 (1218, 1288)	1263 (1228, 1299)	1.01 (0.97, 1.05)
Inpatient care	(34.8)	(35.4)	1.02 (0.99, 1.04)
*Visits (N)*	1.0 (1.0, 1.0)	1.0 (1.0, 1.0)	1.00 (0.99, 1.02)
*Length of stay (N)*	5.7 (5.6, 5.8)	5.6 (5.5, 5.7)	0.98 (0.96, 1.00)
*FFS Sample Spending ($)*	5142 (4848, 5454)	6110 (5760, 6482)	1.19 (1.09, 1.29)
Primary care	(100.0)	(100.0)	1.00 (1.00, 1.00)
*Visits (N)*	18.8 (18.6, 18.9)	15.3 (15.2, 15.4)	0.81 (0.81, 0.82)
*FFS Sample Spending ($)*	2778 (2743, 2814)	1942 (1917, 1967)	0.70 (0.69, 0.71)
Other outpatient care	(93.2)	(94.7)	1.02 (1.01, 1.02)
*Visits (N)*	47.0 (46.6, 47.3)	49.4 (49.1, 49.7)	1.05 (1.04, 1.06)
*FFS Sample Spending ($)*	3606 (3540, 3673)	4176 (4100, 4253)	1.16 (1.13, 1.19)
Prescription drugs	(95.8)	(95.8)	1.00 (1.00, 1.00)
*Filled prescriptions (N)*	45.8 (45.5, 46.0)	47.5 (47.3, 47.8)	1.04 (1.03, 1.05)
*FFS Sample Spending ($)*	4145 (4075, 4216)	4776 (4695, 4857)	1.15 (1.12, 1.18)
Addiction treatment	(47.8)	(49.8)	1.04 (1.02, 1.06)
*Visits (N)*	23.8 (23.5, 24.2)	25.8 (25.4, 26.2)	1.08 (1.06, 1.11)
*FFS Sample Spending ($)*	1479 (1416, 1545)	1462 (1400, 1527)	0.99 (0.93, 1.05)
Total			
*FFS Sample Spending ($)*	16934 (16742, 17128)	18267 (18060, 18477)	1.08 (1.06, 1.10)

FQHC patients who were enrolled in a fee-for-service plan for all Medicaid eligible months also continued to have similar or lower mean spending for emergency department care (IRR = 1.01, 95% CI: 0.97, 1.05), inpatient care (IRR = 1.19, 95% CI: 1.09, 1.29), other outpatient care (IRR = 1.16, 95% CI: 1.13, 1.19), prescription drugs (IRR = 1.15, 95% CI: 1.12, 1.18), and total health care (IRR = 1.08, 95% CI: 1.06, 1.10). FQHC patients also had similar addiction treatment spending (IRR = 0.99, 95% CI: 0.93, 1.05) as compared to non-FQHC patients in the adjusted analysis.

### MOUD access and retention in care

Table 3 compares utilization of MOUD and behavioral health therapies among FQHC patients and non-FQHC patients. A value of less than one reflects lower non-FQHC utilization. MOUD variables are defined as at least one filled prescription or claim for MOUD treatment following an OUD diagnosis. Behavioral health therapy is defined as at least one claim for behavioral health therapy following an OUD diagnosis.

**Table 3:** Use of MOUD and behavioral health therapy among patients with at least six months of continuous Medicaid enrollment after OUD diagnosis by primary care setting: United States, 2012.

Variable	FQHC	Non-FQHC				
		All Non-FQHC		Hospital Outpatient	Physician Office	Mixed Use
	Adjusted (%)	Adjusted (%)	Adjusted RR (CI)	Adjusted RR (CI)	Adjusted RR (CI)	Adjusted RR (CI)
MOUD *≤*90 days of OUD diagnosis						
*Buprenorphine*	(1.3)	(1.5)	1.18 (1.02, 1.36)	1.50 (1.24, 1.80)	1.19 (1.02, 1.38)	0.98 (0.80, 1.19)
*Naltrexone*	(0.9)	(0.7)	0.81 (0.67, 0.98)	0.72 (0.55, 0.93)	0.83 (0.68, 1.03)	0.90 (0.70, 1.16)
*Suboxone*	(10.1)	(11.2)	1.11 (1.05, 1.16)	0.82 (0.77, 0.88)	1.38 (1.31, 1.46)	1.04 (0.97, 1.11)
*Methadone (oral)*	(22.8)	(24.3)	1.07 (1.04, 1.10)	0.98 (0.93, 1.03)	1.07 (1.04, 1.11)	1.08 (1.03, 1.12)
*Any MOUD*	(36.2)	(39.7)	1.10 (1.07, 1.12)	0.97 (0.94, 1.01)	1.18 (1.15, 1.21)	1.07 (1.04, 1.10)
MOUD >180 days of OUD diagnosis						
*Buprenorphine*	(1.0)	(1.2)	1.14 (0.97, 1.34)	1.35 (1.09, 1.67)	1.19 (1.00, 1.41)	1.00 (0.80, 1.24)
*Naltrexone*	(0.6)	(0.4)	0.73 (0.58, 0.93)	0.68 (0.50, 0.93)	0.75 (0.58, 0.97)	0.75 (0.54, 1.03)
*Suboxone*	(9.2)	(10.1)	1.10 (1.04, 1.15)	0.86 (0.80, 0.92)	1.36 (1.29, 1.44)	1.00 (0.93, 1.08)
*Methadone (oral)*	(20.3)	(22.4)	1.10 (1.07, 1.15)	0.99 (0.94, 1.05)	1.12 (1.08, 1.16)	1.09 (1.05, 1.14)
*Any MOUD*	(31.8)	(35.5)	1.12 (1.09, 1.14)	0.98 (0.94, 1.02)	1.20 (1.17, 1.24)	1.07 (1.04, 1.11)
Behavioral Health Therapy *≤*90 days of OUD diagnosis						
*Mental Health*	(29.3)	(26.0)	0.88 (0.86, 0.91)	0.86 (0.82, 0.89)	0.89 (0.86, 0.92)	0.89 (0.86, 0.92)
*Substance Use*	(13.4)	(12.4)	0.93 (0.89, 0.97)	0.94 (0.88, 1.00)	0.94 (0.89, 0.98)	0.96 (0.91, 1.02)
*Any Therapy*	(33.4)	(30.1)	0.90 (0.88, 0.92)	0.88 (0.85, 0.92)	0.92 (0.89, 0.94)	0.90 (0.87, 0.94)
Behavioral Health Therapy >180 days of OUD diagnosis						
*Mental Health*	(24.0)	(21.7)	0.91 (0.88, 0.94)	0.86 (0.82, 0.90)	0.92 (0.89, 0.95)	0.88 (0.84, 0.92)
*Substance Use*	(10.0)	(9.4)	0.93 (0.89, 0.98)	0.93 (0.87, 1.01)	0.94 (0.89, 0.99)	0.96 (0.90, 1.03)
*Any Therapy*	(26.6)	(24.4)	0.92 (0.89, 0.94)	0.88 (0.84, 0.92)	0.94 (0.91, 0.97)	0.89 (0.85, 0.93)

More specifically, as shown in S3.2 Table in S3 File, Unadjusted Analyses, most patients who received MOUD treatment at the time of the study were prescribed methadone or suboxone. Approximately 37% of FQHC patients and 37% of non-FQHC patients received any form of MOUD in the unadjusted analysis. FQHC patients were less likely to receive timely (≤90 days after OUD diagnosis) buprenorphine (FQHC = 1.2% versus non-FQHC = 2.1%) or suboxone (FQHC = 9.8% versus non-FQHC = 13.9%) and more likely to receive timely methadone as compared to non-FQHC patients (FQHC = 24.4.% versus non-FQHC = 18.4%). Access to naltrexone was similar for both groups (FQHC = 0.8% versus non-FQHC = 0.8%). FQHC patients were similarly or slightly more likely to remain in treatment (retention in treatment >180 days after OUD diagnosis) when their MOUD was methadone (FQHC = 21.5% versus non-FQHC = 16.8%) or naltrexone (FQHC = 0.6% versus non-FQHC = 0.5%), but less likely to be retained in treatment when their medication was buprenorphine (FQHC = 1.0% versus non-FQHC = 1.6%) or suboxone (FQHC = 9.0% versus non-FQHC = 12.3%) as compared to non-FQHC patients.

However, in the adjusted analysis, shown in Table 3, FQHC patients were less likely to receive timely buprenorphine (RR = 1.18, 95% CI: 1.02, 1.36), suboxone (RR = 1.11, 95% CI: 1.05, 1.16), or methadone (RR = 1.07, 95% CI: 1.04, 1.10) or be retained in treatment for those MOUDs as compared to non-FQHC patients. FQHC patients were slightly more likely to receive timely naltrexone (RR = 0.81, 95% CI: 0.67, 0.98) and remain in treatment (RR = 0.73, 95% CI: 0.58, 0.93) as compared to non-FQHC patients. Overall, non-FQHC patients were more likely to access any MOUD treatment (RR = 1.10, 95% CI: 1.07, 1.12) and more likely to be being retained in treatment for any MOUD compared to FQHC patients (RR = 1.12, 95% CI: 1.09, 1.14).

We found a more nuanced pattern when we examined non-FQHC patients across care setting by hospital outpatient clinic, physician office, or mixed use (shown in Table 3). Non-FQHC patients who received care in physician offices were more likely to receive timely MOUD (RR = 1.18, 95% CI: 1.15, 1.21) and remain in treatment (RR = 1.20, 95% CI: 1.17, 1.24), with the exception of timely (RR = 0.83, 95% CI: 0.68, 1.03) and continued use of naltrexone (RR = 0.75, 95% CI: 0.58, 0.97). Patients who received the majority of their primary care in hospital outpatient settings were similarly or less likely to receive timely MOUD or be retained in medication treatment as compared to FQHC patients, with the exception of timely (RR = 1.50, 95% CI: 1.24, 1.80) and continued use of buprenorphine (RR = 1.35, 95% CI: 1.09, 1.67).

### Behavioral health therapy access and retention in care

Approximately one-third of FQHC patients (33.5%) and one-quarter of non-FQHC patients (26.2%) received any form of behavioral health therapy, defined as mental health or substance use treatment services, in the unadjusted analysis (S3 File, Unadjusted Analyses). The adjusted analyses in Table 3 further demonstrate FQHC patients were more likely to receive timely (≤90 days) mental health services (RR = 0.88, 95% CI: 0.86, 0.91) or substance use services (RR = 0.93, 95% CI: 0.89, 0.97) and be retained in mental health (RR = 0.91, 95% CI: 0.88, 0.94) or substance use (RR = 0.93, 95% 0.89, 0.98) treatment for more than 180 days. Overall, FQHC patients were more likely to receive any behavioral health therapy (RR = 0.90, 95% CI: 0.88, 0.92) and more likely to remain in behavioral health treatment (RR = 0.92, 95% CI: 0.89, 0.94). Moreover, when FQHCs were compared to specific non-FQHC settings (hospital outpatient, physician offices, or mixed use), FQHC patients were still more likely to receive timely and continued use of behavioral health therapy than patients in any other non-FQHC setting.

### Potentially inappropriate co-prescribing of benzodiazepines and opioids

Table 4 compares FQHC and non-FQHC settings in terms of the number of patients who filled at least one prescription for benzodiazepines, opioid analgesics, or both within 180 days following an OUD diagnosis. A value of less than one reflects lower non-FQHC utilization. The adjusted analysis demonstrates FQHC patients were significantly less likely to fill potentially inappropriate prescriptions for both benzodiazepines and opioid analgesics after OUD diagnosis as compared to non-FQHC patients (RR = 1.35, 95% CI: 1.30, 1.40). Overall, the adjusted results indicate FQHC patients had lower prevalence of potentially inappropriate use of benzodiazepines (FQHC = 25.6% versus non-FQHC = 31.2%), opioid analgesics (FQHC = 39.5% versus non-FQHC = 45.5%), or both (FQHC = 14.8% versus non-FQHC = 20.0%).

**Table 4:** Opioid analgesic and benzodiazepine prescribing among patients with at least six months of continuous Medicaid enrollment after OUD diagnosis by primary care setting: United States, 2012.

Variable	FQHC	Non-FQHC				
		All Non-FQHC		Hospital Outpatient	Physician Office	Mixed Use
	Adjusted (%)	Adjusted (%)	Adjusted RR (CI)	Adjusted RR (CI)	Adjusted RR (CI)	Adjusted RR (CI)
Filled ≥1 benzodiazepine prescription within 180 days after OUD diagnosis	(25.6)	(31.2)	1.22 (1.19, 1.25)	1.03 (0.99, 1.07)	1.34 (1.30, 1.38)	1.12 (1.08, 1.17)
Filled ≥1 opioid analgesic prescription within 180 days after OUD diagnosis	(39.5)	(45.5)	1.15 (1.13, 1.17)	1.07 (1.04, 1.10)	1.21 (1.19, 1.24)	1.17 (1.13, 1.20)
Filled ≥1 benzodiazepine prescription and ≥1 opioid analgesic prescription within 180 days after OUD diagnosis	(14.8)	(20.0)	1.35 (1.30, 1.40)	1.09 (1.03, 1.15)	1.50 (1.44, 1.56)	1.25 (1.19, 1.32)

We also observed notable variation within the non-FQHC group. Physician office patients were significantly more likely than others to exhibit potentially inappropriate co-prescribing. As compared to FQHC patients, hospital outpatient patients were more likely to exhibit potentially inappropriate co-prescribing (RR = 1.09, CI 95%: 1.03, 1.15), but markedly less likely than those receiving care in mixed use settings (RR = 1.25, 95% CI: 1.19, 1.32) or physician offices (RR = 1.50, CI 95%: 1.44, 1.56).

## Discussion

Our results provide a national picture of the experience of Medicaid patients diagnosed with OUD at a time when opioid deaths were increasing and stigma was high [17, 18]. In this nationwide study of a subpopulation of Medicaid patients who were diagnosed with OUD and who received at least one primary care visit in 2012, just over one-third of FQHC patients (37%) and non-FQHC patients (37%) received any form of MOUD. At the time of the study, most patients received methadone or suboxone. Approximately one-third of FQHC patients (33.5%) and one-quarter of non-FQHC patients (26.2%) received mental health or substance use therapy.

Increasing access to primary care has been a key policy strategy to improve access to OUD treatment in the United States [6]. However, there has been limited focus on the ways primary care setting programs and policies have the potential to shape access to OUD treatment and retention in care. We believe that this study is the first to examine health care use and spending among Medicaid patients diagnosed with OUD who receive most of their primary care at FQHCs, which typically adopt an integrated care model, as compared to other primary care settings, including hospital outpatient clinics and physician offices. We noted a number of key differences in patient characteristics across primary care settings, including age, gender, race/ethnicity, geographic region, and dominant Medicaid eligibility group, among others. This underscores the importance of considering the association between primary care setting and health care access, utilization, and quality for Medicaid patients.

Using the propensity score-based overlap weighting method to balance covariates, we found that Medicaid patients who received most of their primary care at FQHCs displayed similar or lower levels of use and spending in all service categories with the exception of higher use and fee-for-service spending on primary care as compared to Medicaid patients who received the majority of their primary care from other settings. In particular, FQHC and non-FQHC patients had similar outcomes for potential opioid-related acute care utilization, defined as emergency department utilization (any past year use and total number of visits) or inpatient hospitalization (any past year use, number of visits, and total inpatient days). When OUD treatment was analyzed in subcategories of MOUD and behavioral health therapy (mental health or substance use services), FQHC patients were more likely to receive timely and continued behavioral health therapy, but less likely to receive timely and continued MOUD. FQHC patients were also less likely to fill potentially inappropriate prescriptions of benzodiazepines and opioid analgesics than non-FQHC patients, particularly patients receiving care in physician offices.

These findings suggest that principal primary care setting may affect outpatient OUD treatment access, quality, and retention in care and merits further exploration to better inform policy and clinical practice. For example, observed patterns suggest that the holistic and integrated health care model adopted by many FQHCs may improve access to behavioral health therapies as well as retention in care. By 2013, approximately 38% of FQHCs had achieved or maintained Patient-Centered Medical Home recognition, a model of care promoting comprehensive, patient-centered, coordinated care with accessible services focused on improving quality and safety [19, 20]. Since that time, the number of FQHCs using this model has more than doubled. By June 2020, 78% of all HRSA-funded FQHCs, supporting patients diagnosed with OUD by enabling same-day visits to specialists in the same clinic location or creating warm-handoffs or referrals.

The advantages of coordinated care in FQHCs may also be reflected in the lower prevalence of potentially inappropriate co-prescribing of benzodiazepines and opioid analgesics among FQHC patients as compared to patients receiving the majority of their primary care from other settings. Much of the overall difference between the FQHC and non-FQHC patient groups arose within the physician-office group. FQHCs may benefit from advances in health information technology that many physician offices lack. These systems enable advanced functionality including clinical decision support, clinical information exchange, and electronic prescribing. They facilitate tracking of patient medical and pharmacy records in an integrated electronic health record system to understand which prescriptions are contraindicated while managing multiple comorbidities and different providers and specialists, including in 2012, when approximately 80% of all FQHCs had an electronic health record installed for all sites and providers. Further, it may be easier for FQHCs to implement and enforce common organizational best-practices and to monitor and discourage poor practices (such as co-prescribing benzodiazepines or opioids analgesics for patients diagnosed with OUD) than can be done within a decentralized system that relies on individual providers.

More research is needed to understand whether and why FQHC patients appeared less likely to receive MOUD as compared to non-FQHC patients after adjusting for measurable confounding between patient groups. The prescription of controlled substances for MOUDs is highly regulated in all health care settings by the FDA, the Drug Enforcement Administration (DEA), and the Substance Abuse and Mental Health Services Administration (SAMHSA). Prescribers must obtain a waiver under the Drug Addiction Treatment Act (DATA) prior to the prescribing of buprenorphine, suboxone, methadone, or other combination products for the treatment of OUD. While we do not have data on the number of FQHC providers with DATA waivers for 2012, FQHCs began reporting this data to HRSA in 2017, demonstrating progress towards improving access to MOUD for FQHC patients in recent years. Between 2017 and 2019, the number of FQHCs that reported providing MOUD to patients increased from 472 (34%) to 803 (58%) FQHCs [21]. The number of providers (physicians, physician assistants, and certified nurse practitioners) increased 139% from 2,973 to 7,095 and patients increased 121% from 64,597 to 142,919 patients [21].

### Limitations

Our study findings should be evaluated in light of several limitations. The most complete Medicaid claims data available for national analysis of all 50 states and the District of Columbia were for the year 2012. This is due to variations in the timing of state transitions from the MAX data system to Transformed Medicaid Statistical Information System (T-MSIS) Analytic Files (TAF) between 2013–2015 and across-state and within-state variations in the quality of the TAF data, the most recent Medicaid data available for all states [22]. This limited our ability to analyze the effects of the Affordable Care Act Medicaid expansions that began in 2014 and the rapid evolution and intensification of the OUD epidemic since 2015. Nevertheless, our data did allow us to examine the effects of principal primary care setting on MOUD utilization, OUD treatment quality, and other health care utilization and spending among a larger number of Medicaid patients in a greater number of states than previous studies.

There have also been notable advances in primary care and OUD treatment in recent years. For example, utilization of electronic medical record systems has improved since 2012 from 79% to 98% among FQHCs, which may enhance prospects for integrated care. Further, between 2014 and 2019, HRSA awarded nearly $1 billion to expand access to mental health services and substance use disorder treatment [23–27]. These investments have resulted in marked increases in OUD treatment services, including MOUD, according to annual data reported through the Uniform Data System [28]. Finally, there is now greater awareness of the dangers of prescription opioids and inappropriate co-prescribing than there was in 2012. Best-practice guidelines promulgated by the Centers for Disease Control and Prevention (CDC) and others have influenced practice across all care settings. Since we did not examine site-specific or provider-specific patterns with these data, a minority of physicians may account for a disproportionate share of contraindicated care practices, such as co-prescribing.

Finally, we note standard limitations of propensity score based weighting methods. Although we can account for differences in observed variables, we cannot account for differences in unobserved patient characteristics, preferences, and circumstances that could lead some patients to receive care at FQHCs. Future work should examine how organizational factors and variations in state-level policies and programs affect health care access, utilization, and quality of care for Medicaid patients diagnosed with OUD.

## Conclusions

Examining a critical early phase of the opioid epidemic, our nationwide analyses demonstrated key benefits associated with the integrated care environment of FQHCs as well as areas that appeared to require continued process improvement. Medicaid patients diagnosed with OUD who received the majority of their primary care at FQHCs displayed higher primary care utilization and fee-for-service spending, yet similar or lower utilization and fee-for-service spending for other health service categories. Further, Medicaid patients diagnosed with OUD who received most primary care at FQHCs, were less likely to have potentially inappropriate prescriptions filled, and were more likely to receive behavioral health therapies as compared to patients who received most primary care in other settings. At the same time, FQHCs lagged somewhat behind in the provision of the most common forms of MOUD. The broad pattern of our research underscores the need to consider differences in health care settings in efforts to address the opioid epidemic nationwide.

## Supporting information

S1 File Data availability statement and construction. (DOCX)

S2 File Propensity overlap weighting balancing table. (DOCX)

S3 File Unadjusted analyses. (DOCX)

S4 File Sensitivity analyses. (DOCX)
