# Supplementary material for: Health care use and spending for Medicaid patients diagnosed with opioid use disorder receiving primary care in Federally Qualified Health Centers and other primary care settings
Source: PLoS One. 2022 Oct 18;17(10):e0276066. doi: 10.1371/journal.pone.0276066 (PMC9578596; doi:10.1371/journal.pone.0276066)
Supplement: S1 File — (DOCX) [file pone.0276066.s001.docx]

**Supporting information**

**S1 File. Data Availability Statement and Construction**

**Data Availability Statement**

We accessed 2012 Medicaid Analytic eXtract (MAX) data through a Data Use Agreement (DUA) with the Centers for Medicare and Medicaid Services. Since the data includes protected health information and personally identifiable, we are unable to share this third party data. However, we detail how we constructed our data sample and variables for other researchers with access to Medicaid claims data.

**Construction of Opioid Use Disorder**

###

### **OUD Diagnosis**

International Classification of Disease Ninth Revision (ICD-9) codes used to identify opioid use disorder (OUD) diagnosis were selected based on a review of prior research.^[[1]](#footnote-1),^^[[2]](#footnote-2),^^[[3]](#footnote-3),^^[[4]](#footnote-4)^

We used codes 304.00 – 304.03, 304.70 – 304.73, 305.50 – 305.53, 965.00 – 965.02, and 965.09 in any position on at least one claim to identify patients diagnosed with OUD in 2012. Most patients in our sample had a diagnosis of opioid dependence - unspecified (304.00) or opioid dependence – continuous (304.01). Less than 20% of patients in our sample had a diagnosis of opioid abuse (305.50 – 305.53) or opioid poisoning (965.00 – 965.02 and 965.09).

**Construction of General Spending and Utilization Outcomes**

Building on methodologies developed by Mukamel et al. (2015) and Nocon et al. (2016), we used 2012 claim-level data in the Medicaid Analytic Extract (MAX) files to construct individual-level health utilization outcomes.^[[5]](#footnote-5)^,^[[6]](#footnote-6)^

Utilization outcomes were identified using the Current Procedural Terminology (CPT) codes, type of service, and place of service codes. Health Care Provider Taxonomy codes were also used to identify addiction treatment visits. Spending for each type of utilization summed total payments from Medicaid as well as third-party payers.

### **Addiction Treatment Visits**

Addiction treatment was defined as services provided by an addiction treatment specialist. We identified addiction treatment specialists using Health Care Provider Taxonomy Codes.

### **Emergency Room Visits**

All claims associated with one emergency room visit were collapsed into that emergency room visit. Outpatient claims that overlapped with an emergency room visit were also collapsed into that emergency room visit.

### **Inpatient Visits**

All claims associated with an inpatient stay were collapsed into the inpatient visit.

### **Primary Care Visits**

Primary care visits were identified using the following CPT codes: 99201– 99205, 99211 – 99215, 99391 – 99397, and 99381– 99387. Visits without these CPT codes, but billed by providers with primary care related taxonomy codes and provided at primary care settings were also classified as primary care visits.

### **Other Outpatient Care Visits**

Nonprimary outpatient care visits were differentiated from primary care visits based on CPT codes, types of service, and places of service codes.

### **Prescription Drugs**

Filled prescription drugs were identified using National Drug Code (NDC) codes.

**Construction of Opioid Use Disorder Treatment Outcomes**

### **Medication for Opioid Use Diagnosis (MOUD)**

We used the Substance Use and Mental Health Services Administration (SAMHSA) definition for Medication for Opioid Use Disorder (MOUD), which includes buprenorphine, naltrexone, methadone, and suboxone (buprenorphine/naloxone).^[[7]](#footnote-7)^ Drawing on clinical expertise and the existing literature^[[8]](#footnote-8)^, we identified pharmacy claims for buprenorphine (without naloxone), suboxone, and naltrexone with the aid of an NDC list compiled using National Library of Medicine's RxNorm API. We used Healthcare Common Procedure Coding System (HCPCS) codes in Other Services (OT) to identify methadone (H0020, J1230) and oral or long-acting injectable naltrexone (J2315, T1502).

### **Behavioral Health Therapy**

To identify behavioral health therapy codes, we reviewed the CPT and Healthcare Common Procedure Coding System (HCPCS) codes used by Cole et al. (2019), Hadland et al. (2018), and McCarty et al. (2019). We categorized relevant codes used in those studies as mental health or substance use treatment and reviewed the categorizations with clinicians.

**Mental Health Codes:** 90791 – 90792, 90832 – 90834, 90836 – 90840, 90853, 90863, 90875 – 90876, 99401 – 99404, 99411 – 99412, 99420, 99429, 99455 – 99456, H0004, H0031, H0036 – H0037, H0039 – H0040, H2000 – H2001, H2011 – H2020, S9480, S9484 – S9485

**Substance Use Codes:** G0396 – G0397, H0001 – H0002, H0005 – H0007, H0014 – H0016, H0020, H0022, H0028, H0034, H0047, H0050, H2010, H2035 – H2036, M0064, T1006, T1012

### **Benzodiazepine and Opioid Analgesic Potentially Inappropriate Co-Prescribing**

Drawing on research by Stein et al. (2017) as well as clinical and pharmaceutical expertise, we used NDC codes to identify benzodiazepine and opioid analgesic (other than buprenorphine) prescriptions filled following an OUD diagnosis in 2012.^[[9]](#footnote-9)^

***Benzodiazepines:*** Alprazolam, Chlordiazepoxide, Clobazam, Clonazepam, Clorazepate, Diazepam, Estazolam, Flurazepam, Lorazepam, Oxazepam, Quazepam, Temazepam, Triazolam

***Opioid Analgesics:*** Fentanyl, Hydrocodone, Hydromorphone, Levorphanol, Meperidine, Methadone, Morphine, Opium, Oxycodone, Oxymorphone, Pentazocine, Tapentadol, Tramadol

1. Cole, E. S., DiDomenico, E., Cochran, G., Gordon, A. J., Gellad, W. F., Pringle, J., ... & Kelley, D. (2019). The role of primary care in improving access to medication-assisted treatment for rural Medicaid enrollees with opioid use disorder. *Journal of General Internal Medicine*, *34*(6), 936-943. [↑](#footnote-ref-1)
2. McCarty, D., Gu, Y., McIlveen, J. W., & Lind, B. K. (2019). Medicaid expansion and treatment for opioid use disorders in Oregon: an interrupted time-series analysis. *Addiction science & clinical practice*, *14*(1), 31. [↑](#footnote-ref-2)
3. Young, K., & Zur, J. (2017). Medicaid and the opioid epidemic: enrollment, spending, and the implications of proposed policy changes. *The Henry J. Kaiser Family Foundation*. [↑](#footnote-ref-3)
4. Hadland, S. E., Bagley, S. M., Rodean, J., Silverstein, M., Levy, S., Larochelle, M. R., ... & Zima, B. T. (2018). Receipt of timely addiction treatment and association of early medication treatment with retention in care among youths with opioid use disorder. *JAMA pediatrics*, *172*(11), 1029-1037. [↑](#footnote-ref-4)
5. Mukamel, D. B., White, L. M., Nocon, R. S., Huang, E. S., Sharma, R., Shi, L., & Ngo‐Metzger, Q. (2016). Comparing the cost of caring for Medicare beneficiaries in federally funded FQHCs to other care settings. *Health services research*, *51*(2), 625-644. [↑](#footnote-ref-5)
6. Nocon, R. S., Lee, S. M., Sharma, R., Ngo-Metzger, Q., Mukamel, D. B., Gao, Y., ... & Huang, E. S. (2016). Health care use and spending for Medicaid enrollees in federally qualified health centers versus other primary care settings. *American journal of public health*, *106*(11), 1981-1989. [↑](#footnote-ref-6)
7. Substance Abuse and Mental Health Services Administration. TIP 63: Medications for Opioid Use Disorder. Rockville: Substance Abuse and Mental Health Services Administration; 2018. Accessed at https://store.samhsa.gov/product/TIP-63-Medications-for-Opioid-Use-Disorder-Full-Document/PEP20-02-01-006 on 22 November 2020. [↑](#footnote-ref-7)
8. McCarty, D., Gu, Y., McIlveen, J. W., & Lind, B. K. (2019). Medicaid expansion and treatment for opioid use disorders in Oregon: an interrupted time-series analysis. *Addiction science & clinical practice*, *14*(1), 31. [↑](#footnote-ref-8)
9. Stein, B. D., Mendelsohn, J., Gordon, A. J., Dick, A. W., Burns, R. M., Sorbero, M., ... & Liccardo Pacula, R. (2017). Opioid analgesic and benzodiazepine prescribing among Medicaid-enrollees with opioid use disorders: The influence of provider communities. *Journal of addictive diseases*, *36*(1), 14-22. [↑](#footnote-ref-9)
