# Supplementary material for: Health care use and spending for Medicaid patients diagnosed with opioid use disorder receiving primary care in Federally Qualified Health Centers and other primary care settings
Source: PLoS One. 2022 Oct 18;17(10):e0276066. doi: 10.1371/journal.pone.0276066 (PMC9578596; doi:10.1371/journal.pone.0276066)
Supplement: S2 File — (DOCX) [file pone.0276066.s002.docx]

**S2 File. Propensity Overlap Weighting Balancing Table**

**Table S2.1 Comparison of Federally Qualified Health Center (FQHC) and Non-FQHC Patient Characteristics after Matching Using Propensity Score Overlap Weighting: All States**

| **Characteristics /**  **Covariates used for matching** | **Crude** | | | | | **Overlap Weight Method** | | | | |
| --- | --- | --- | --- | --- | --- | --- | --- | --- | --- | --- |
|  | **FQHC**  **(N=37412)**  **N (%) or Mean ± SD** | **Non-FQHC**  **(N=196712)**  **N (%) or Mean ± SD** | **Mean Difference** | **Standard Error of Mean Difference** | **Standardized Difference** | **FQHC**  **(N=37412)**  **N (%) or Mean ± SD** | **Non-FQHC**  **(N=196712)**  **N (%) or Mean ± SD** | **Mean Difference** | **Standard Error of Mean Difference** | **Standardized Difference** |
| Age (years) |  ±** ** |  ±  | 2.58 | 11.52 | 22.4 |  ±  |  ±  | 0 | 7.52 | 0 |
| Female | 18707 (50.4) | 114008 (58.0) | -7.59 | 0.50 | 15.28 | 13531 (51.3) | 13531 (51.3) | 0 | 0.50 | 0 |
| Race/ethnicity  *White*  *Black*  *Hispanic or Hispanic and >1 Race*  *Asian or Pacific Islander*  *American Indian or Alaska Native*  *Native Hawaiian or Pacific* *Islander*  *Non-Hispanic and >1 Race*  *Missing* | 19633 (52.9)  7366 (19.8)  6865 (18.5)  168 (0.5)  493 (1.3)  122 (0.3)  162 (0.4)  2333 (6.3) | 128973 (65.6)  28084 (14.3)  22912 (11.6)  898 (0.5)  2632 (1.3)  326 (0.2)  1049 (0.5)  11838 (6.0) | -12.71  5.56  6.84  0.00  -0.01  0.16  -0.10  0.26 | 0.49  0.38  0.36  0.07  0.11  0.05  0.07  0.24 | 26.07  14.81  19.20  0.06  0.09  3.28  1.40  1.10 | 14611 (55.4)  4618 (17.5)  4730 (17.9)  131 (0.5)  340 (1.3)  83 (0.3)  131 (0.5)  1742 (6.6) | 14611 (55.4)  4618 (17.5)  4730 (17.9)  131 (0.5)  340 (1.3)  83 (0.3)  131 (0.5)  1742 (6.6) | 0  0  0  0  0  0  0  0 | 0.50  0.38  0.38  0.07  0.11  0.06  0.07  0.25 | 0  0  0  0  0  0  0  0 |
| Dominant Medicaid eligibility group  *Blind/disabled*  *Adult*  *State demonstration* | 15205 (41.0)  13529 (36.4)  8408 (22.6) | 68441 (34.8)  80293 (40.8)  47978 (24.4) | 6.14  -4.39  -1.75 | 0.48  0.49  0.42 | 12.69  9.03  4.13 | 10334 (39.2)  9275 (35.2)  6774 (25.7) | 10334 (39.2)  9275 (35.2)  6774 (25.7) | 0  0  0 | 0.49  0.48  0.44 | 0  0  0 |
| Medicaid eligible months | 11.6 ±  |  ±  | 0.04 | 1.15 | 3.65 | 11.6 ±  |  ±  | 0 | 0.75 | 0 |
| Medicaid managed care months |  ±  |  ±  | -1.89 | 5.25 | 36.07 | 6.4 ±  | 6.4 ±  | 0 | 3.55 | 0 |
| Census Region  *Midwest*  *Northeast*  *South*  *West* | 3746 (10.1)  19752 (53.2)  4950 (13.3)  8694 (23.4) | 40074 (20.4)  90228 (45.9)  39337 (20.0)  27073 (13.8) | -10.29  7.31  -6.67  9.64 | 0.36  0.50  0.37  0.39 | 28.93  14.66  17.97  24.99 | 3254 (12.3)  14735 (55.8)  2645 (10.0)  5750 (21.8) | 3254 (12.3)  14735 (55.8)  2645 (10.0)  5750 (21.8) | 0  0  0  0 | 0.33  0.50  0.30  0.41 | 0  0  0  0 |
| Urban | 33238 (89.5) | 162424 (82.6) | 6.92 | 0.34 | 20.06 | 23329 (88.4) | 23329 (88.4) | 0 | 0.32 | 0 |
| Distance from FQHC (km) |  ±  |  ±  | -8.00 | 19.93 | 40.14 | 11.4 ±  |  ±  | 0 | 10.68 | 0 |
| TANF eligible | 2309 (6.2) | 12572 (6.4) | -0.17 | 0.24 | 0.72 | 1760 (6.7) | 1760 (6.7) | 0 | 0.25 | 0 |
| Elixhauser risk score |  ±  |  ±  | 0.10 | 2.42 | 4.03 | 3.5 ±  |  ±  | 0 | 1.54 | 0 |

**Table S2.1 Comparison of Federally Qualified Health Center (FQHC) and Non-FQHC Patient Characteristics after Matching Using Propensity Score Overlap Weighting: All States (Continued)^[[1]](#footnote-1)^**

| **Characteristics /**  **Covariates used for matching (State)** | **Crude** | | | | | **Overlap Weight Method** | | | | |
| --- | --- | --- | --- | --- | --- | --- | --- | --- | --- | --- |
|  | **FQHC**  **(N=37412)**  **N (%) or Mean ± SD** | **Non-FQHC**  **(N=196712)**  **N (%) or Mean ± SD** | **Mean Difference** | **Standard Error of Mean Difference** | **Standardized Difference** | **FQHC**  **(N=37412)**  **N (%) or Mean ± SD** | **Non-FQHC**  **(N=196712)**  **N (%) or Mean ± SD** | **Mean Difference** | **Standard Error of Mean Difference** | **Standardized Difference** |
| Alabama (AL) | 49 (0.1) | 1598 (0.8) | -0.59 | 0.07 | 8.21 | 78 (0.3) | 78 (0.3) | 0 | 0.05 | 0 |
| Alaska (AK) | 83 (0.2) | 421 (0.2) | -0.08 | 0.04 | 1.98 | 44 (0.2) | 44 (0.2) | 0 | 0.04 | 0 |
| Arkansas (AR) | -- | -- | -- | -- | -- | -- | -- | -- | -- | -- |
| Arizona (AZ) | 312 (0.8) | 6320 (3.2) | -2.37 | 0.14 | 16.9 | 294 (1.1) | 294 (1.1) | 0 | 0.10 | 0 |
| California (CA) | 5197 (14.0) | 7938 (4.0) | 9.96 | 0.28 | 35.31 | 3079 (11.7) | 3079 (11.7) | 0 | 0.32 | 0 |
| Colorado (CO) | 258 (0.7) | 990 (0.5) | 0.19 | 0.08 | 2.48 | 203 (0.8) | 203 (0.8) | 0 | 0.09 | 0 |
| Connecticut (CT) | 5100 (13.7) | 6223 (3.2) | 10.57 | 0.27 | 38.7 | 2726 (10.3) | 2726 (10.3) | 0 | 0.30 | 0 |
| District of Columbia (DC) | 382 (1.0) | 824 (0.4) | 0.61 | 0.08 | 7.2 | 260 (1.0) | 260 (1.0) | 0 | 0.10 | 0 |
| Delaware (DE) | <11 (<0.01) | 3202 (1.6) | -1.63 | 0.09 | 18.15 | <11 (0.0) | <11 (0.0) | 0 | 0.01 | 0 |
| Florida (FL) | 294 (0.8) | 4590 (2.3) | -1.54 | 0.12 | 12.46 | 274 (1.0) | 274 (1.0) | 0 | 0.10 | 0 |
| Georgia (GA) | 119 (0.3) | 1756 (0.9) | -0.57 | 0.08 | 7.38 | 109 (0.4) | 109 (0.4) | 0 | 0.06 | 0 |
| Hawaii (HI) | 245 (0.7) | 546 (0.3) | 0.38 | 0.07 | 5.6 | 171 (0.6) | 171 (0.6) | 0 | 0.08 | 0 |
| Idaho (ID) | 33 (0.1) | 337 (0.2) | -0.08 | 0.04 | 2.29 | 30 (0.1) | 30 (0.1) | 0 | 0.03 | 0 |
| Iowa (IA) | 69 (0.2) | 518 (0.3) | -0.08 | 0.05 | 1.64 | 60 (0.2) | 60 (0.2) | 0 | 0.05 | 0 |
| Illinois (IL) | 1371 (3.7) | 5296 (2.7) | 1.00 | 0.18 | 5.69 | 1070 (4.1) | 1070 (4.1) | 0 | 0.20 | 0 |
| Indiana (IN) | 195 (0.5) | 2354 (1.2) | -0.67 | 0.09 | 7.28 | 180 (0.7) | 180 (0.7) | 0 | 0.08 | 0 |
| Kansas (KS) | 40 (0.1) | 393 (0.2) | -0.09 | 0.04 | 2.35 | 35 (0.1) | 35 (0.1) | 0 | 0.04 | 0 |
| Kentucky (KY) | 127 (0.3) | 3261 (1.7) | -1.32 | 0.10 | 13.25 | 122 (0.5) | 122 (0.5) | 0 | 0.07 | 0 |
| Louisiana (LA) | 98 (0.3) | 1345 (0.7) | -0.42 | 0.07 | 6.12 | 91 (0.3) | 91 (0.3) | 0 | 0.06 | 0 |
| Massachusetts (MA) | 4024 (10.8) | 21389 (10.9) | -0.04 | 0.31 | 0.13 | 3193 (12.1) | 3193 (12.1) | 0 | 0.33 | 0 |
| Maryland (MD) | 2795 (7.5) | 1075 (0.5) | 6.98 | 0.19 | 36.03 | 758 (2.9) | 758 (2.9) | 0 | 0.17 | 0 |
| Maine (ME) | 1206 (3.2) | 4628 (2.4) | 0.89 | 0.16 | 5.42 | 943 (3.6) | 943 (3.6) | 0 | 0.19 | 0 |
| Michigan (MI) | 503 (1.4) | 6698 (3.4) | -2.05 | 0.15 | 13.49 | 465 (1.8) | 465 (1.8) | 0 | 0.13 | 0 |
| Minnesota (MN) | 108 (0.3) | 5887 (3.0) | -2.70 | 0.13 | 21.38 | 105 (0.4) | 105 (0.4) | 0 | 0.06 | 0 |
| Missouri (MO) | 155 (0.4) | 1386 (0.7) | -0.29 | 0.07 | 3.85 | 133 (0.5) | 133 (0.5) | 0 | 0.07 | 0 |
| Mississippi (MS) | 36 (0.1) | 960 (0.5) | -0.39 | 0.05 | 7.25 | 35 (0.1) | 35 (0.1) | 0 | 0.04 | 0 |

**Table S2.1 Comparison of Federally Qualified Health Center (FQHC) and Non-FQHC Patient Characteristics after Matching Using Propensity Score Overlap Weighting: All States (Continued)**

| **Characteristics /**  **Covariates used for matching (State)** | **Crude** | | | | | **Overlap Weight Method** | | | | |
| --- | --- | --- | --- | --- | --- | --- | --- | --- | --- | --- |
|  | **FQHC**  **(N=37412)**  **N (%) or Mean ± SD** | **Non-FQHC**  **(N=196712)**  **N (%) or Mean ± SD** | **Mean Difference** | **Standard Error of Mean Difference** | **Standardized Difference** | **FQHC**  **(N=37412)**  **N (%) or Mean ± SD** | **Non-FQHC**  **(N=196712)**  **N (%) or Mean ± SD** | **Mean Difference** | **Standard Error of Mean Difference** | **Standardized Difference** |
| Montana (MT) | 39 (0.1) | 264 (0.1) | -0.03 | 0.03 | 0.84 | 33 (0.1) | 33 (0.1) | 0 | 0.04 | 0 |
| North Carolina (NC) | 111 (0.3) | 6332 (3.2) | -2.92 | 0.13 | 22.35 | 109 (0.4) | 109 (0.4) | 0 | 0.06 | 0 |
| North Dakota (ND) | <11 (<0.01) | 110 (0.1) | -0.04 | 0.02 | 2.28 | <11 (0.0) | <11 (0.0) | 0 | 0.01 | 0 |
| Nebraska (NE) | <11 (<0.01) | 138 (0.1) | -0.06 | 0.02 | 3.14 | <11 (0.0) | <11 (0.0) | 0 | 0.01 | 0 |
| Nevada (NV) | 32 (0.1) | 732 (0.4) | -0.29 | 0.05 | 5.98 | 26 (0.1) | 26 (0.1) | 0 | 0.03 | 0 |
| New Hampshire (NH) | 131 (0.4) | 1057 (0.5) | -0.18 | 0.07 | 2.77 | 116 (0.4) | 116 (0.4) | 0 | 0.07 | 0 |
| New Jersey (NJ) | 1006 (2.7) | 7102 (3.6) | -0.90 | 0.17 | 5.16 | 884 (3.3) | 884 (3.3) | 0 | 0.18 | 0 |
| New Mexico (NM) | 256 (0.7) | 1854 (0.9) | -0.25 | 0.09 | 2.82 | 223 (0.8) | 223 (0.8) | 0 | 0.09 | 0 |
| New York (NY) | 7048 (19.0) | 41892 (21.3) | -2.32 | 0.40 | 5.79 | 5865 (22.2) | 5865 (22.2) | 0 | 0.42 | 0 |
| Ohio (OH) | 816 (2.2) | 12024 (6.1) | -3.92 | 0.20 | 19.72 | 761 (2.9) | 761 (2.9) | 0 | 0.17 | 0 |
| Oklahoma (OK) | 55 (0.1) | 1990 (1.0) | -0.86 | 0.08 | 11.39 | 53 (0.2) | 53 (0.2) | 0 | 0.04 | 0 |
| Oregon (OR) | 1170 (3.2) | 3685 (1.9) | 1.28 | 0.16 | 8.17 | 860 (3.3) | 860 (3.3) | 0 | 0.18 | 0 |
| Pennsylvania (PA) | 238 (0.6) | 4010 (2.0) | -1.40 | 0.11 | 12.18 | 221 (0.8) | 221 (0.8) | 0 | 0.09 | 0 |
| Rhode Island (RI) | 334 (0.9) | 1580 (0.8) | 0.10 | 0.09 | 1.05 | 281 (1.1) | 281 (1.1) | 0 | 0.10 | 0 |
| South Carolina (SC) | 92 (0.2) | 1214 (0.6) | -0.37 | 0.07 | 5.63 | 85 (0.3) | 85 (0.3) | 0 | 0.06 | 0 |
| South Dakota (SD) | <11 (<0.01) | 47 (<0.01) | -0.01 | 0.01 | 1.00 | <11 (0.0) | <11 (0.0) | 0 | 0.01 | 0 |
| Tennessee (TN) | 121 (0.3) | 4998 (2.5) | -2.21 | 0.12 | 18.72 | 118 (0.4) | 118 (0.4) | 0 | 0.07 | 0 |
| Texas (TX) | 162 (0.4) | 2301 (1.2) | -0.73 | 0.09 | 8.23 | 151 (0.6) | 151 (0.6) | 0 | 0.08 | 0 |
| Utah (UT) | 38 (0.1) | 1311 (0.7) | -0.56 | 0.06 | 9.13 | 37 (0.1) | 37 (0.1) | 0 | 0.04 | 0 |
| Virginia (VA) | 79 (0.2) | 1995 (1.0) | -0.80 | 0.08 | 10.28 | 75 (0.3) | 75 (0.3) | 0 | 0.05 | 0 |
| Vermont (VT) | 665 (1.8) | 2347 (1.2) | 0.60 | 0.12 | 4.93 | 506 (1.9) | 506 (1.9) | 0 | 0.14 | 0 |
| Washington (WA) | 1065 (2.9) | 2590 (1.3) | 1.55 | 0.14 | 10.85 | 751 (2.8) | 751 (2.8) | 0 | 0.17 | 0 |
| West Virginia (WV) | 395 (1.1) | 1896 (1.0) | 0.10 | 0.10 | 0.99 | 327 (1.2) | 327 (1.2) | 0 | 0.11 | 0 |
| Wisconsin (WI) | 477 (1.3) | 5223 (2.7) | -1.37 | 0.14 | 9.88 | 433 (1.6) | 433 (1.6) | 0 | 0.13 | 0 |
| Wyoming (WY) | <11 (0.0) | 85 (<0.01) | -0.04 | 0.01 | 2.94 | <11 (0.0) | <11 (0.0) | 0 | 0.00 | 0 |

1. We do not report the exact number of patients diagnosed with OUD in any category with less than 11 patients in accordance with our data use agreement. [↑](#footnote-ref-1)
