# Supplementary material for: Health care use and spending for Medicaid patients diagnosed with opioid use disorder receiving primary care in Federally Qualified Health Centers and other primary care settings
Source: PLoS One. 2022 Oct 18;17(10):e0276066. doi: 10.1371/journal.pone.0276066 (PMC9578596; doi:10.1371/journal.pone.0276066)
Supplement: S3 File — (DOCX) [file pone.0276066.s003.docx]

**S3 File. Unadjusted Analyses**

**Table S3.1. Unadjusted Annual Use and Spending for FQHC OUD Patients Compared with Non-FQHC OUD Patients with at least Six Methods of Continuous Medicaid Enrollment after OUD Diagnosis: United States, 2012**

| **Annual Utilization or Spending** | **FQHC (N = 37142),**  **N (%) or Mean ± SD** | **Non-FQHC (N = 196712),**  **N (%) or Mean ± SD** | **Adjusted RR / IRR**^[[1]](#footnote-1)^**,**  **(95% CI)** |
| --- | --- | --- | --- |
| Emergency department  *Visits (N)*  *FFS Sample Spending ($)^[[2]](#footnote-2)^* | (66.5)  3.2 ± 6.3  1241 ± 3575 | (66.5)  3.2 ± 6.4  1344 ± 5098 | 0.99 (0.98, 1.00)  0.98 (0.97, 0.99)  1.01 (0.97, 1.05) |
| Inpatient care  *Visits (N)*  *Length of stay (N)*  *FFS Sample Spending ($)* | (33.9)  1.0 ± 2.9  5.7 ± 15.7  5099 ± 17730 | (36.5)  1.0 ± 2.5  5.6 ± 15.3  6423 ± 21216 | 1.02 (0.99, 1.04)  1.00 (0.99, 1.02)  0.98 (0.96, 1.00)  1.19 (1.09, 1.29) |
| Primary care  *Visits (N)*  *FFS Sample Spending ($)* | (100.0)  17.8 ± 28.7  2792 ± 4720 | (100.0)  16.1 ± 26.2  1979 ± 3718 | 1.00 (1.00, 1.00)  0.81 (0.81, 0.82)  0.70 (0.69, 0.71) |
| Other outpatient care^[[3]](#footnote-3)^  *Visits (N)*  *FFS Sample Spending ($)* | (93.6)  47.9 ± 65.1  3712 ± 6655 | (94.0)  46.5 ± 74.0  4064 ± 9679 | 1.02 (1.01, 1.02)  1.05 (1.04, 1.06)  1.16 (1.13, 1.19) |
| Prescription drugs  *Filled prescriptions (N)*  *FFS Sample Spending ($)* | (95.8)  45.8 ± 44.2  4279 ± 7957 | (96.3)  47.1 ± 44.7  4543 ± 11376 | 1.00 (1.00, 1.00)  1.04 (1.03, 1.05)  1.15 (1.12, 1.18) |
| Addiction treatment  *Visits (N)*  *FFS Sample Spending ($)* | (48.0)  24.1 ± 50.8  1617 ± 2785 | (42.4)  21.6 ± 53.0  1143 ± 2715 | 1.04 (1.02, 1.06)  1.08 (1.06, 1.11)  0.99 (0.93, 1.05) |
| Total  *FFS Sample Spending ($)* | 17124 ± 24512 | 18353 ± 30480 | 1.08 (1.06, 1.10) |

**Table S3.2 Use of MOUD and Behavioral Health Therapy among Patients with at Least Six Months of Continuous Medicaid Enrollment after OUD Diagnosis by Primary Care Setting: United States, 2012**

| **Variable** | **FQHC**  **(N = 37142)** | **Non-FQHC (N = 196712)** | | | |
| --- | --- | --- | --- | --- | --- |
|  |  | **All Non-FQHC**  **(N = 196712)** | **Hospital Outpatient**  **(N = 51053)** | **Physician Office**  **(N = 114940)** | **Mixed Use**  **(N = 30719)** |
|  | Unadjusted  (%) | Unadjusted  (%) | Unadjusted  (%) | Unadjusted  (%) | Unadjusted  (%) |
| MOUD *≤*90 days of OUD diagnosis^[[4]](#footnote-4)^  *Buprenorphine*  *Naltrexone*  *Suboxone*  *Methadone (oral)*  *Any MOUD* | (1.2)  (0.8)  (9.8)  (24.4)  (37.3) | (2.1)  (0.8)  (13.9)  (18.4)  (36.7) | (1.6)  (0.6)  (9.9)  (11.9)  (25.6) | (2.3)  (0.8)  (16.1)  (19.8)  (40.7) | (2.0)  (0.9)  (12.1)  (23.8)  (40.3) |
| MOUD >180 days of OUD diagnosis  *Buprenorphine*  *Naltrexone*  *Suboxone*  *Methadone (oral)*  *Any MOUD* | (1.0)  (0.6)  (9.0)  (21.5)  (32.8) | (1.6)  (0.5)  (12.3)  (16.8)  (32.3) | (1.2)  (0.4)  (9.3)  (11.3)  (23.4) | (1.7)  (0.5)  (14.2)  (18.1)  (35.6) | (1.5)  (0.5)  (10.4)  (21.3)  (34.6) |
| Behavioral Health Therapy *≤*90 days of OUD diagnosis ^[[5]](#footnote-5)^  *Mental Health*  *Substance Use*  *Any Therapy* | (29.5)  (13.0)  (33.5) | (21.1)  (12.3)  (26.2) | (15.3)  (7.4)  (18.5) | (22.5)  (13.8)  (28.4) | (25.7)  (14.6)  (30.9) |
| Behavioral Health Therapy >180 days of OUD diagnosis  *Mental Health*  *Substance Use*  *Any Therapy* | (24.0)  (9.6)  (26.5) | (17.8)  (9.4)  (20.9) | (13.7)  (6.1)  (15.9) | (18.9)  (10.4)  (22.5) | (20.3)  (10.8)  (23.5) |

**Table S3.3 Opioid Analgesic and Benzodiazepine Prescribing among Patients with at Least Six Months of Continuous Medicaid Enrollment after OUD Diagnosis by Primary Care Setting: United States, 2012**

| **Variable** | **FQHC**  **(N = 37142)** | **Non-FQHC (N = 196712)** | | | |
| --- | --- | --- | --- | --- | --- |
|  |  | **All Non-FQHC**  **(N = 196712)** | **Hospital Outpatient**  **(N = 51053)** | **Physician Office**  **(N = 114940)** | **Mixed Use**  **(N = 30719)** |
|  | Unadjusted  (%) | Unadjusted  (%) | Unadjusted  (%) | Unadjusted  (%) | Unadjusted  (%) |
| Filled ≥1 benzodiazepine prescription within 180 days after OUD diagnosis | (25.4) | (32.2) | (25.1) | (35.7) | (30.7) |
| Filled ≥1 opioid analgesic prescription within 180 days after OUD diagnosis | (40.2) | (47.5) | (38.5) | (51.2) | (48.5) |
| Filled ≥1 benzodiazepine prescription and ≥1 opioid analgesic prescription within 180 days after OUD diagnosis | (14.8) | (21.2) | (14.1) | (24.5) | (20.6) |

1. Note: The ratios compare the adjusted non-FQHC estimate to the adjusted FQHC estimate. A value of less than one reflects lower non-FQHC utilization or spending. IRR with 95% CI is presented for count variables with means. RR with 95% CI is presented for binary variables with percentages. [↑](#footnote-ref-1)
2. Note: FFS spending estimates only include patients with FFS coverage for all eligible months due to concerns with quality of managed care expenditure data in some states. The FFS populations include 14,708 FQHC patients and 44,820 non-FQHC patients. Additional data is included in the Appendix. [↑](#footnote-ref-2)
3. Note: Other outpatient care is defined as non-primary care, non-transportation services, and non-dental outpatient claims. [↑](#footnote-ref-3)
4. Note: Defined as at least one filled prescription or claim for MOUD treatment following OUD diagnosis. See Appendix for NDC codes. [↑](#footnote-ref-4)
5. Note: Defined as at least one claim for behavioral health therapy following OUD diagnosis. See Appendix for procedure codes. [↑](#footnote-ref-5)
