# Supplementary material for: Health care use and spending for Medicaid patients diagnosed with opioid use disorder receiving primary care in Federally Qualified Health Centers and other primary care settings
Source: PLoS One. 2022 Oct 18;17(10):e0276066. doi: 10.1371/journal.pone.0276066 (PMC9578596; doi:10.1371/journal.pone.0276066)
Supplement: S4 File — (DOCX) [file pone.0276066.s004.docx]

**S4. Sensitivity Analysis**

### **Table S4.1 Timely Use of MOUD among Patients with at Least Six Months of Continuous Medicaid Enrollment after OUD Diagnosis by Primary Care Setting (FQHC and Non-FQHC): United States, 2012**

| **Variable** | **FQHC**  **(N = 37142)** | | **Non-FQHC**  **(N = 196712)** | | **Adjusted**  **IRR / RR^[[1]](#footnote-1)^ (CI)** |
| --- | --- | --- | --- | --- | --- |
|  | Unadjusted  (%) | Adjusted  (%) | Unadjusted  (%) | Adjusted  (%) |  |
| MOUD *≤*30 days of OUD diagnosis^[[2]](#footnote-2)^  *Buprenorphine*  *Naltrexone*  *Suboxone*  *Methadone (oral)*  *Any MOUD* | (0.9)  (0.5)  (8.2)  (23.3)  (34.1) | (1.0)  (0.5)  (8.4)  (21.8)  (32.9) | (1.6)  (0.5)  (11.7)  (17.7)  (33.1) | (1.2)  (0.4)  (9.5)  (23.5)  (36.4) | 1.16 (0.99, 1.37)  0.87 (0.68, 1.11)  1.12 (1.06, 1.19)  1.08 (1.04, 1.11)  1.11 (1.08, 1.13) |
| MOUD *≤*60 days of OUD diagnosis  *Buprenorphine*  *Naltrexone*  *Suboxone*  *Methadone (oral)*  *Any MOUD* | (1.1)  (0.7)  (9.1)  (23.9)  (36.0) | (1.2)  (0.7)  (9.5)  (22.3)  (34.9) | (1.9)  (0.6)  (13.0)  (18.1)  (35.3) | (1.4)  (0.6)  (10.5)  (23.9)  (38.4) | 1.14 (0.98, 1.32)  0.81 (0.66, 1.00)  1.11 (1.06, 1.17)  1.07 (1.04, 1.11)  1.10 (1.08, 1.13) |
| MOUD *≤*90 days of OUD diagnosis  *Buprenorphine*  *Naltrexone*  *Suboxone*  *Methadone (oral)*  *Any MOUD* | (1.2)  (0.8)  (9.8)  (24.4)  (37.3) | (1.3)  (0.9)  (10.1)  (22.8)  (36.2) | (2.1)  (0.8)  (13.9)  (18.4)  (36.7) | (1.5)  (0.7)  (11.2)  (24.3)  (39.7) | 1.18 (1.02, 1.36)  0.81 (0.67, 0.98)  1.11 (1.05, 1.16)  1.07 (1.04, 1.10)  1.10 (1.07, 1.12) |
| MOUD *≤*180 days of OUD diagnosis  *Buprenorphine*  *Naltrexone*  *Suboxone*  *Methadone (oral)*  *Any MOUD* | (1.4)  (1.1)  (11.4)  (25.5)  (40.3) | (1.5)  (1.2)  (11.9)  (23.8)  (39.1) | (2.5)  (1.0)  (16.1)  (19.2)  (39.9) | (1.8)  (0.9)  (13.0)  (25.4)  (42.6) | 1.18 (1.04, 1.35)  0.79 (0.67, 0.93)  1.09 (1.05, 1.14)  1.07 (1.03, 1.10)  1.09 (1.07, 1.11) |

### **Table S4.2 Continuous Use of MOUD among Patients with at Least Six Months of Continuous Medicaid Enrollment after OUD Diagnosis by Primary Care Setting (FQHC and Non-FQHC): United States, 2012**

| **Variable** | **FQHC**  **(N = 37142)** | | **Non-FQHC**  **(N = 196712)** | | **Adjusted**  **IRR / RR^[[3]](#footnote-3)^ (CI)** |
| --- | --- | --- | --- | --- | --- |
|  | Unadjusted  (%) | Adjusted  (%) | Unadjusted  (%) | Adjusted  (%) |  |
| MOUD >30 days of OUD diagnosis^[[4]](#footnote-4)^  *Buprenorphine*  *Naltrexone*  *Suboxone*  *Methadone (oral)*  *Any MOUD* | (1.5)  (1.2)  (12.4)  (25.8)  (41.4) | (1.6)  (1.3)  (12.8)  (24.1)  (40.2) | (2.7)  (1.0)  (17.2)  (19.8)  (41.2) | (2.0)  (1.0)  (13.9)  (25.8)  (43.8) | 1.26 (1.11, 1.44)  0.76 (0.64, 0.89)  1.09 (1.04, 1.13)  1.07 (1.04, 1.10)  1.09 (1.07, 1.11) |
| MOUD >60 days of OUD diagnosis  *Buprenorphine*  *Naltrexone*  *Suboxone*  *Methadone (oral)*  *Any MOUD* | (1.4)  (1.0)  (11.8)  (25.3)  (40.1) | (1.5)  (1.1)  (12.2)  (23.6)  (38.9) | (2.5)  (0.9)  (16.4)  (19.3)  (39.8) | (1.8)  (0.9)  (13.3)  (25.3)  (42.5) | 1.26 (1.10, 1.44)  0.78 (0.65, 0.93)  1.09 (1.04, 1.14)  1.07 (1.04, 1.10)  1.09 (1.07, 1.11) |
| MOUD >90 days of OUD diagnosis  *Buprenorphine*  *Naltrexone*  *Suboxone*  *Methadone (oral)*  *Any MOUD* | (1.3)  (0.9)  (11.3)  (24.7)  (38.9) | (1.4)  (1.0)  (11.7)  (23.1)  (37.7) | (2.3)  (0.8)  (15.7)  (18.8)  (38.5) | (1.7)  (0.8)  (12.7)  (24.8)  (41.3) | 1.22 (1.06, 1.39)  0.79 (0.65, 0.95)  1.09 (1.04, 1.14)  1.07 (1.04, 1.11)  1.10 (1.07, 1.12) |
| MOUD >180 days of OUD diagnosis  *Buprenorphine*  *Naltrexone*  *Suboxone*  *Methadone (oral)*  *Any MOUD* | (1.0)  (0.6)  (9.0)  (21.5)  (32.8) | (1.0)  (0.6)  (9.2)  (20.3)  (31.8) | (1.6)  (0.5)  (12.3)  (16.8)  (32.3) | (1.2)  (0.4)  (10.1)  (22.4)  (35.5) | 1.14 (0.97, 1.34)  0.73 (0.58, 0.93)  1.10 (1.04, 1.15)  1.10 (1.07, 1.15)  1.12 (1.09, 1.14) |

### **Table S4.3 Timely Use of Behavioral Health Therapy among Patients with at Least Six Months of Continuous Medicaid Enrollment after OUD Diagnosis by Primary Care Setting (FQHC and Non-FQHC): United States, 2012**

| **Variable** | **FQHC**  **(N = 37142)** | | **Non-FQHC**  **(N = 196712)** | | **Adjusted**  **IRR / RR^[[5]](#footnote-5)^ (CI)** |
| --- | --- | --- | --- | --- | --- |
|  | Unadjusted  (%) | Adjusted  (%) | Unadjusted  (%) | Adjusted  (%) |  |
| Behavioral Health Therapy *≤*30 days of OUD diagnosis^[[6]](#footnote-6)^  *Mental Health*  *Substance Use*  *Any Therapy* | (23.7)  (9.7)  (27.7) | (23.3)  (10.1)  (27.5) | (15.9)  (9.7)  (21.1) | (20.6)  (9.6)  (25.0) | 0.88 (0.86, 0.91)  0.95 (0.90, 1.00)  0.91 (0.88, 0.93) |
| Behavioral Health Therapy *≤*60 days of OUD diagnosis  *Mental Health*  *Substance Use*  *Any Therapy* | (27.2)  (11.7)  (31.2) | (26.9)  (12.1)  (31.0) | (19.0)  (11.2)  (24.1) | (23.8)  (11.3)  (28.0) | 0.88 (0.86, 0.91)  0.94 (0.89, 0.98)  0.90 (0.88, 0.92) |
| Behavioral Health Therapy *≤*90 days of OUD diagnosis  *Mental Health*  *Substance Use*  *Any Therapy* | (29.5)  (13.0)  (33.5) | (29.3)  (13.4)  (33.4) | (21.1)  (12.3)  (26.2) | (25.9)  (12.4)  (30.1) | 0.88 (0.86, 0.91)  0.93 (0.89, 0.97)  0.90 (0.88, 0.92) |
| Behavioral Health Therapy *≤*180 days of OUD diagnosis  *Mental Health*  *Substance Use*  *Any Therapy* | (34.1)  (15.7)  (38.2) | (34.2)  (16.1)  (38.4) | (25.7)  (14.7)  (31.0) | (30.2)  (14.6)  (34.5) | 0.88 (0.86, 0.91)  0.91 (0.87, 0.94)  0.90 (0.88, 0.92) |

### **Table S4.4 Continuous Use of Behavioral Health Therapy among Patients with at Least Six Months of Continuous Medicaid Enrollment after OUD Diagnosis by Primary Care Setting (FQHC and Non-FQHC): United States, 2012**

| **Variable** | **FQHC**  **(N = 37142)** | | **Non-FQHC**  **(N = 196712)** | | **Adjusted**  **IRR / RR^[[7]](#footnote-7)^ (CI)** |
| --- | --- | --- | --- | --- | --- |
|  | Unadjusted  (%) | Adjusted  (%) | Unadjusted  (%) | Adjusted  (%) |  |
| Behavioral Health Therapy >30 days of OUD diagnosis^[[8]](#footnote-8)^  *Mental Health*  *Substance Use*  *Any Therapy* | (35.7)  (16.1)  (39.4) | (35.8)  (16.5)  (39.6) | (27.6)  (15.0)  (32.2) | (31.7)  (14.8)  (35.5) | 0.89 (0.86, 0.91)  0.89 (0.86, 0.93)  0.90 (0.88, 0.92) |
| Behavioral Health Therapy >60 days of OUD diagnosis  *Mental Health*  *Substance Use*  *Any Therapy* | (33.8)  (14.7)  (37.3) | (33.9)  (15.2)  (37.4) | (25.9)  (13.9)  (30.3) | (30.1)  (13.7)  (33.6) | 0.89 (0.86, 0.91)  0.90 (0.86, 0.94)  0.90 (0.88, 0.92) |
| Behavioral Health Therapy >90 days of OUD diagnosis  *Mental Health*  *Substance Use*  *Any Therapy* | (31.4)  (13.5)  (34.6) | (31.6)  (13.9)  (34.9) | (24.1)  (12.8)  (28.2) | (28.1)  (12.7)  (31.5) | 0.89 (0.87, 0.92)  0.91 (0.87, 0.95)  0.90 (0.88, 0.92) |
| Behavioral Health Therapy >180 days of OUD diagnosis  *Mental Health*  *Substance Use*  *Any Therapy* | (24.0)  (9.6)  (26.5) | (24.0)  (10.0)  (26.6) | (17.8)  (9.4)  (20.9) | (21.7)  (9.4)  (24.4) | 0.91 (0.88, 0.97)  0.93 (0.89, 0.98)  0.92 (0.89, 0.94) |

### **Table S4.5 Opioid Analgesic and Benzodiazepine Prescribing among Patients with at Least Six Months of Continuous Medicaid Enrollment after OUD Diagnosis by Primary Care Setting (FQHC and Non-FQHC): United States, 2012**

| **Variable** | **FQHC**  **(N = 37142)** | | **Non-FQHC**  **(N = 196712)** | | **Adjusted**  **IRR / RR^[[9]](#footnote-9)^ (CI)** |
| --- | --- | --- | --- | --- | --- |
|  | Unadjusted  (%) | Adjusted  (%) | Unadjusted  (%) | Adjusted  (%) |  |
| Filled ≥1 benzodiazepine prescription within 30 days after OUD diagnosis | (17.0) | (17.1) | (22.1) | (21.6) | 1.26 (1.22, 1.31) |
| Filled ≥1 opioid analgesic prescription within 30 days after OUD diagnosis | (21.2) | (21.0) | (28.3) | (26.8) | 1.28 (1.24, 1.32) |
| Filled ≥1 benzodiazepine prescription and ≥1 opioid analgesic prescription within 30 days after OUD diagnosis | (6.9) | (7.0) | (10.9) | (10.3) | 1.48 (1.40, 1.56) |
| Filled ≥1 benzodiazepine prescription within 60 days after OUD diagnosis | (19.9) | (20.1) | (25.8) | (25.0) | 1.25 (1.21, 1.29) |
| Filled ≥1 opioid analgesic prescription within 60 days after OUD diagnosis | (27.2) | (26.8) | (34.4) | (32.7) | 1.22 (1.19, 1.25) |
| Filled ≥1 benzodiazepine prescription and ≥1 opioid analgesic prescription within 60 days after OUD diagnosis | (9.3) | (9.4) | (14.2) | (13.4) | 1.43 (1.36, 1.50) |
| Filled ≥1 benzodiazepine prescription within 90 days after OUD diagnosis | (21.6) | (21.8) | (27.9) | (27.1) | 1.24 (1.21, 1.28) |
| Filled ≥1 opioid analgesic prescription within 90 days after OUD diagnosis | (31.2) | (30.8) | (38.7) | (36.8) | 1.20 (1.17, 1.22) |
| Filled ≥1 benzodiazepine prescription and ≥1 opioid analgesic prescription within 90 days after OUD diagnosis | (10.9) | (11.0) | (16.4) | (15.4) | 1.41 (1.35, 1.47) |
| Filled ≥1 benzodiazepine prescription within 180 days after OUD diagnosis | (25.4) | (25.6) | (32.2) | (31.2) | 1.22 (1.19, 1.25) |
| Filled ≥1 opioid analgesic prescription within 180 days after OUD diagnosis | (40.2) | (39.5) | (47.5) | (45.5) | 1.15 (1.13, 1.17) |
| Filled ≥1 benzodiazepine prescription and ≥1 opioid analgesic prescription within 180 days after OUD diagnosis | (14.8) | (14.8) | (21.2) | (20.0) | 1.35 (1.30, 1.40) |

1. The ratios compare the adjusted non-FQHC estimate to the adjusted FQHC estimate. A value of less than one reflects lower non-FQHC utilization or spending. IRR with 95% CI is presented for count variables with means. RR with 95% CI is presented for binary variables with percentages. [↑](#footnote-ref-1)
2. Defined as at least one filled prescription or claim for MOUD treatment following OUD diagnosis. [↑](#footnote-ref-2)
3. The ratios compare the adjusted non-FQHC estimate to the adjusted FQHC estimate. A value of less than one reflects lower non-FQHC utilization or spending. IRR with 95% CI is presented for count variables with means. RR with 95% CI is presented for binary variables with percentages. [↑](#footnote-ref-3)
4. Defined as at least one filled prescription or claim for MOUD treatment following OUD diagnosis. [↑](#footnote-ref-4)
5. The ratios compare the adjusted non-FQHC estimate to the adjusted FQHC estimate. A value of less than one reflects lower non-FQHC utilization or spending. IRR with 95% CI is presented for count variables with means. RR with 95% CI is presented for binary variables with percentages. [↑](#footnote-ref-5)
6. Defined as at least one claim for behavioral health therapy following OUD diagnosis. [↑](#footnote-ref-6)
7. The ratios compare the adjusted non-FQHC estimate to the adjusted FQHC estimate. A value of less than one reflects lower non-FQHC utilization or spending. IRR with 95% CI is presented for count variables with means. RR with 95% CI is presented for binary variables with percentages. [↑](#footnote-ref-7)
8. Defined as at least one claim for behavioral health therapy following OUD diagnosis. [↑](#footnote-ref-8)
9. The ratios compare the adjusted non-FQHC estimate to the adjusted FQHC estimate. A value of less than one reflects lower non-FQHC utilization or spending. IRR with 95% CI is presented for count variables with means. RR with 95% CI is presented for binary variables with percentages. [↑](#footnote-ref-9)
